# Quantification of Lipid-Rich Core in Carotid Atherosclerosis Using Magnetic Resonance T_2_ Mapping: Relation to Clinical Presentation

**DOI:** 10.1016/j.jcmg.2016.06.013

**Published:** 2017-07

**Authors:** Joshua T. Chai, Luca Biasiolli, Linqing Li, Mohammad Alkhalil, Francesca Galassi, Chris Darby, Alison W. Halliday, Linda Hands, Timothy Magee, Jeremy Perkins, Ed Sideso, Ashok Handa, Peter Jezzard, Matthew D. Robson, Robin P. Choudhury

**Affiliations:** a Division of Cardiovascular Medicine, Radcliffe Department of Medicine, University of Oxford, Oxford, United Kingdom; b National Institute of Mental Health, National Institutes of Health, Bethesda, Maryland; c Nuffield Department of Surgical Sciences, University of Oxford, Oxford, United Kingdom; d FMRIB Centre, Nuffield Department of Clinical Neurosciences, University of Oxford, Oxford, United Kingdom

**Keywords:** atherosclerosis, cardiovascular magnetic resonance, carotid, lipid, plaque, T_2_ mapping, AHA, American Heart Association, CMR, cardiovascular magnetic resonance, DANTE, delay alternating with nutation for tailored excitation, IPH, intraplaque hemorrhage, LRNC, lipid-rich necrotic core, MESE, multiecho spin echo, ROC, receiver-operating characteristic, TOF, time-of-flight

## Abstract

**Objectives:**

The aim of this study was to: 1) provide tissue validation of quantitative T_2_ mapping to measure plaque lipid content; and 2) investigate whether this technique could discern differences in plaque characteristics between symptom-related and non–symptom-related carotid plaques.

**Background:**

Noninvasive plaque lipid quantification is appealing both for stratification in treatment selection and as a possible predictor of future plaque rupture. However, current cardiovascular magnetic resonance (CMR) methods are insensitive, require a coalesced mass of lipid core, and rely on multicontrast acquisition with contrast media and extensive post-processing.

**Methods:**

Patients scheduled for carotid endarterectomy were recruited for 3-T carotid CMR before surgery. Lipid area was derived from segmented T_2_ maps and compared directly to plaque lipid defined by histology.

**Results:**

Lipid area (%) on T_2_ mapping and histology showed excellent correlation, both by individual slices (R = 0.85, p < 0.001) and plaque average (R = 0.83, p < 0.001). Lipid area (%) on T_2_ maps was significantly higher in symptomatic compared with asymptomatic plaques (31.5 ± 3.7% vs. 15.8 ± 3.1%; p = 0.005) despite similar degrees of carotid stenosis and only modest difference in plaque volume (128.0 ± 6.0 mm^3^ symptomatic vs. 105.6 ± 9.4 mm^3^ asymptomatic; p = 0.04). Receiver-operating characteristic analysis showed that T_2_ mapping has a good ability to discriminate between symptomatic and asymptomatic plaques with 67% sensitivity and 91% specificity (area under the curve: 0.79; p = 0.012).

**Conclusions:**

CMR T_2_ mapping distinguishes different plaque components and accurately quantifies plaque lipid content noninvasively. Compared with asymptomatic plaques, greater lipid content was found in symptomatic plaques despite similar degree of luminal stenosis and only modest difference in plaque volumes. This new technique may find a role in determining optimum treatment (e.g., providing an indication for intensive lipid lowering or by informing decisions of stents vs. surgery).

## Graphical abstract

**Figure:**
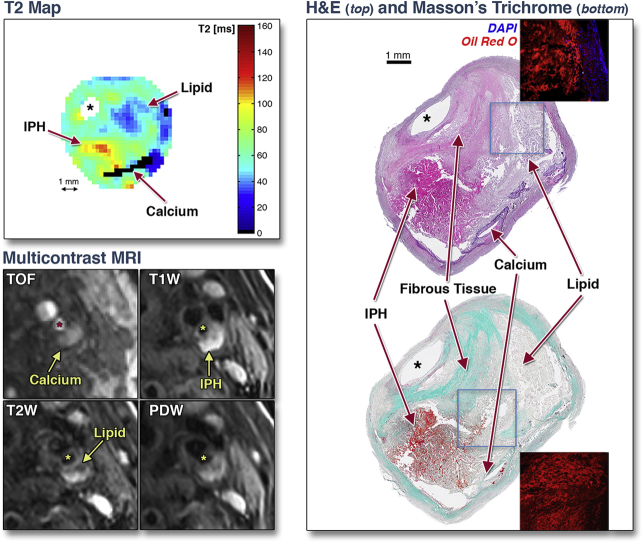

Lipid accumulation in the subendothelial space, following deposition and retention of apolipoprotein B–containing plasma lipoproteins, is a key process in the initiation and progression of atherosclerosis (1). Studies of ex vivo tissue in coronary (2) and carotid (3) atherosclerosis have demonstrated that plaques associated with thrombotic complications tend to contain larger lipid-rich necrotic core (LRNC), among other high-risk features such as thin fibrous cap, infiltration of inflammatory cells, and the presence of intraplaque hemorrhage (IPH). Therefore, 1 of the key goals in risk-stratifying vulnerable plaques has become the identification and quantification of LRNC using in vivo vessel wall imaging (4).

Although intensive lipid-lowering therapy can reduce total carotid vessel wall area (5), direct evidence of lipid removal has been sparse (6). One particular challenge is that evacuated lipids tend to be replaced by fibrous tissue (7), so that changes in total vessel wall area might be small or indiscernible by conventional imaging. As a new generation of lipid-lowering agents emerges 8, 9, tools for robust quantitative assessment of plaque composition may facilitate refinement and stratification for patient selection, and allow better monitoring of treatment response.

Multicontrast cardiovascular magnetic resonance (CMR) is an established technique in plaque characterization (10). However, optimal techniques for quantitative LRNC detection on multicontrast CMR require injection of contrast media 6, 11, and rely on tissue contrast relative to the adjacent sternocleidomastoid muscle, which depends on specific system and acquisition parameters. Moreover, multicontrast CMR suffers from blurring artifacts due to fast spin echo acquisitions (12) and needs extensive post-processing to coregister different contrast-weighted images and correct for image intensity variations (13).

We recently reported a quantitative CMR method to map T_2_ relaxation times of plaque components on a voxel-by-voxel basis (14). Compared to multicontrast CMR, this novel approach is more objective, as it requires minimal user interaction in the analysis, and calculates real quantitative information on plaque composition. This raises the important possibility of in vivo plaque lipid quantification, at high resolution, across the entire plaque, and without the use of gadolinium-based contrast. In addition, plaque T_2_ mapping addresses the need for an absolute physical parameter that can be standardized among different CMR systems and widely adopted in multicenter studies. Accordingly, here we sought: 1) to validate lipid quantification on carotid T_2_ maps using histology gold standard; and 2) to evaluate its potential clinical application in relation to identifying recently unstable plaques.

## Methods

### Study population

Ethical approval was obtained from National Research Ethics Services and local R&D committee. Forty patients awaiting carotid endarterectomy at Oxford University Hospitals NHS Trust were recruited from November 2011 to June 2014. Patients were scanned at the Oxford Acute Vascular Imaging Centre ≤24 h before surgery. Carotid plaques were collected at operation. Patients either had recently symptomatic (median time from index event 2 weeks) or asymptomatic carotid disease, with 50% to 99% stenosis according to NASCET (North American Symptomatic Carotid Endarterectomy Trial), or 70% to 99% according to ESCT (European Carotid Surgery Trial) criteria. Plaques were defined as culprit plaques where they were deemed to have given rise to either a minor cerebrovascular accident or a transient ischemic attack as assessed clinically and supported, where available, by brain magnetic resonance imaging or computed tomography imaging. Asymptomatic carotid plaques were those that had no documented clinical symptoms, but with an indication for carotid endarterectomy on the basis of percent stenosis.

### CMR protocol

Patients were imaged on a Verio 3-T scanner (Siemens Healthcare, Erlangen, Germany) with a 4-channel phased-array carotid coil (Machnet, Roden, the Netherlands). Bright-blood time-of-flight (TOF) angiography of the carotid arteries was acquired to localize carotid bifurcation and lumen stenosis. Two-dimensional carotid T_2_ maps were generated from 14 images with echo time of 9 to 127 ms and repetition time of 2,000 ms, acquired using the multislice delay alternating with nutation for tailored excitation multiecho spin-echo (DANTE-MESE) sequence that combined black-blood preparation on the basis of nonselective DANTE pulse trains (15) with chemical-shift-selective fat saturated MESE. DANTE-MESE acquired 10 slices of 2 mm thickness each, covering 2 cm of the target carotid artery, in 8 min.

### Data analysis

DANTE-MESE image quality was assessed by an experienced reviewer (L.B.) who inspected the visibility of the carotid wall boundaries and rated them from 1 (poor) to 5 (excellent) according to standard procedures (16). Patients with overall quality <3 were excluded from the study.

T_2_ maps of the carotid arteries were generated voxel by voxel using monoexponential nonlinear fitting (14); lumen and external vessel boundaries were segmented using a validated semiautomated procedure (17). As reported previously, LRNC without IPH had shorter T_2_ than normal vessel wall and fibrous tissue, whereas with recent IPH into the core, T_2_ was longer than all other plaque components (14). Therefore, to take into account the minority of plaques with recent hemorrhage into the core, segmentation used dual T_2_ thresholds, a lower one (T_2L_) indicating the maximum T_2_ value associated with lipid alone and a higher one (T_2H_) above which IPH was recorded inside the plaque lipid core. Voxels with T_2_ values below T_2L_ or above T_2H_ were thus classified as LRNC. Significant IPH (T_2_ > T_2H_) was defined as >5% of the total cross-sectional area. We also reported LRNC values on the basis of T_2L_ alone, taking no account of hemorrhage.

To use the data most efficiently, segmentation performance was estimated by leave-1-out cross-validation. Lipid area (%) was computed for all possible combinations of T_2L_ (range 30 to 50 ms) and T_2H_ (range 70 to 120 ms) to find the pair of threshold values that achieved the optimal lipid segmentation on T_2_ maps (i.e., the highest Pearson correlation coefficient R against lipid quantification on histology). All algorithms were implemented in Matlab (MathWorks, Natick, Massachusetts). Plaque type was also classified on the basis of T_2_ mapping and TOF data (for identification of IPH and calcium) using the modified American Heart Association (AHA) scheme (10).

### Histology

Carotid plaques were collected at endarterectomy and divided into 2 halves at the point of maximal stenosis: one half was snap-frozen and the other half processed for paraffin embedding. Paraffin sections (5 μm) were obtained at 1-mm intervals for histological examination using hematoxylin and eosin (Sigma-Aldrich, Poole, United Kingdom) and Masson’s trichrome (Merck-Millipore, Nottingham, United Kingdom), which identified collagen in the fibrous tissue (green), as well as fibrin and erythrocytes from IPH (bright red). Lipid area without previous IPH appeared transparent or unstained in both hematoxylin and eosin and Masson’s trichrome stain. Stained slides were scanned using the NanoZoomer (Hamamatsu Photonics, Hamamatsu City, Japan) virtual pathology system. Frozen sections (20 μm) at the mirror plane of division on 20 randomly selected plaques were obtained for Oil Red O (Sigma-Aldrich) staining to confirm lipid presence. Because IPH occurred exclusively inside, and as part of, the LRNC, the boundary of IPH against LRNC was often impossible to define on histology (Online Figure 1). The total histological lipid area (%) was hence calculated by combining lipid area with and without IPH. Total vessel cross-sectional area and lipid area (%) were obtained by manual segmentation using ImageJ, 64-bit, version 1.46r (National Institutes of Health, Bethesda, Maryland), as previously described (18). Plaque type was classified histologically using the AHA scheme (10).

### Location matching

One investigator (L.B.) blinded to histology measured the distance from carotid bifurcation of T_2_ map locations and provided the corresponding T_1_-weighted images to a second investigator (J.T.C.) who obtained histology on the basis of the specific distance from bifurcation, and fine-tuned the exact matching location using local vessel morphology from the T_1_-weighted images provided.

### Scan-rescan reproducibility

Scan-rescan reproducibility of lipid quantification by T_2_ mapping was studied in 10 additional patients recruited on an unselected sequential basis. They were taken out of the scanner after the first scan and left sitting for 2 min before the second scan. Both scans acquired 10 contiguous slices to cover the entire plaque and assess the reproducibility of lipid area (%) per plaque.

### Statistical analysis

All statistical analyses are reported as mean ± SEM, unless otherwise stated; Student *t* tests and chi-square tests were performed. Lipid area correlation was calculated using both slice locations and plaques as the unit of analysis. Leave-1-out cross-validation was performed on the slice-by-slice dataset of independent lipid area measurements from T_2_ maps and histology.

## Results

### Patient characteristics

CMR scan quality ≥3 was achieved for 26 of 40 plaques, 15 symptomatic and 11 asymptomatic. Patient characteristics are summarized in Table 1. There was no significant difference between genders, major cardiovascular risk factors or medications on admission between groups.

**Table 1:** Summary of Patient Characteristics Between Symptomatic and Asymptomatic Groups

	Symptomatic (n = 15)	Asymptomatic (n = 11)	Significance (p Value)
Male:female ratio	2.75:1	2.67:1	NS
Age, yrs	73 (49–90)	60 (43–89)	0.046
CV risks			
Hypertension	14 (93.3)	9 (81.8)	NS
Hypercholesterolemia	12 (80.0)	7 (63.6)	NS
Smoking	7 (46.7)	4 (36.4)	NS
Diabetes mellitus	4 (26.7)	4 (36.4)	NS
Previous CAD/CVA	4 (26.7)	6 (54.5)	NS
Medication at time of CEA			
Aspirin/antiplatelets	12 (80.0)	8 (72.7)	NS
Statins	14 (93.3)	10 (90.9)	NS
Beta-blockers	4 (26.7)	3 (27.3)	NS
Calcium antagonists	5 (33.3)	3 (27.3)	NS
ACE inhibitors/ARBs	6 (40.0)	5 (45.5)	NS
Anticoagulation	3 (20.0)	0 (0)	NS
Duplex ultrasound scan			
Right:left ratio	6.5:1	1:1.2	0.038
Stenosis, %	81.3 ± 2.5	84.1 ± 3.0	NS

Values are median (range), n (%), or mean ± SEM.

ACE = angiotensin-converting enzyme; ARB = angiotensin receptor blocker; CAD = coronary artery disease; CEA = carotid endarterectomy; CV = cardiovascular; CVA = cerebrovascular accident; NS = not significant.

### AHA classification

**Figure 1:**
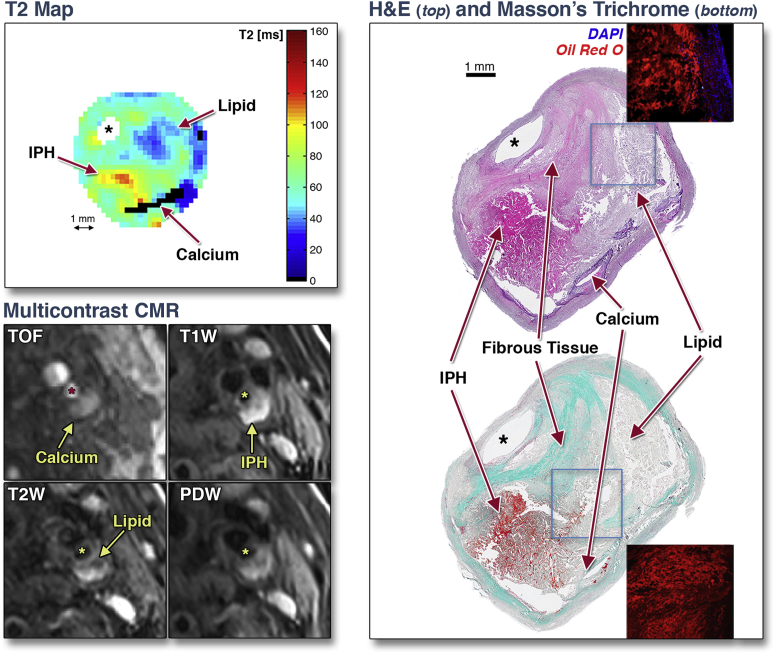
Comparison of T_2_ Map, Multicontrast CMR, and Histology T_2_ mapping identifies different components of an American Heart Association type VI plaque showing the presence of lipid **(blue)**, recent intraplaque hemorrhage (IPH) **(yellow/red)**, and calcium **(black)**. These plaque components are also visible on different weightings of multicontrast cardiovascular magnetic resonance (CMR) and are confirmed by hematoxylin and eosin (H&E) and Masson’s staining on histology. Lipid was further confirmed on Oil Red O staining on adjacent frozen section. **Asterisk** indicates lumen. DAPI = 4',6-diamidino-2-phenylindole fluorescent nuclear stains; PDW = proton density weighted; T1W = T_1_ weighted; T2W = T_2_ weighted; TOF = time-of-flight.

Figure 1 shows how different plaque components can be identified on T_2_ maps. To evaluate how accurately T_2_ mapping can determine plaque types, we used the modified AHA plaque classification system and compared this directly against histological classification. Table 2 shows plaque types determined by T_2_ map (+ TOF) against histology, which showed good agreement (80.8%) between the 2 methods (Cohen’s κ = 0.73).

**Table 2:** Modified AHA Plaque Type Classification by T_2_ Mapping (+ TOF) Versus Histology Showed Good Agreement (80.8%)

Histology	CMR (T2 Maps + TOF)				
	IV to V	VI	VII	VIII	Total
IV to V	7	2	—	—	9
VI	1	7	—	—	8
VII	1	—	4	1	6
VIII	—	—	—	3	3
Total	9	9	4	4	26

Cohen’s κ = 0.73.

AHA = American Heart Association; CMR = cardiovascular magnetic resonance; TOF = time-of-flight.

### Lipid quantification

**Figure 2:**
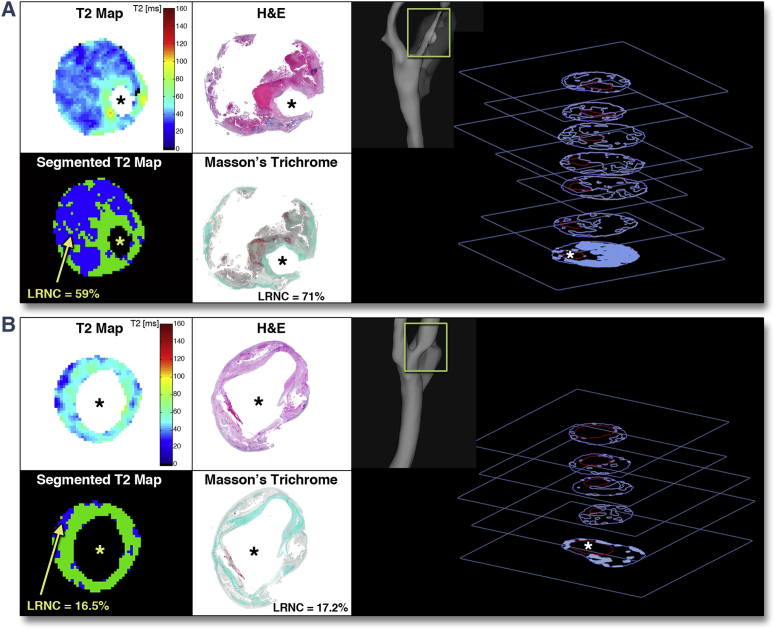
Quantification of Plaque Lipid T_2_ and segmented map of **(A)** a plaque with large lipid-rich necrotic core (LRNC) and **(B)** a plaque with multiple smaller lipid-rich pools, and their matching histology. LRNC was segmented from the T_2_ map by thresholding (voxels with T_2_ <42 ms or T_2_ >90 ms). **Right panels** show 3-dimensional representations of plaque lipid distribution. Stacks of slices show lumen **(red)** and lipid **(blue contours)** segmented from T_2_ maps (for illustration). The * signifies the lumen of the vessel. H&E = hematoxylin and eosin.

**Figure 3:**
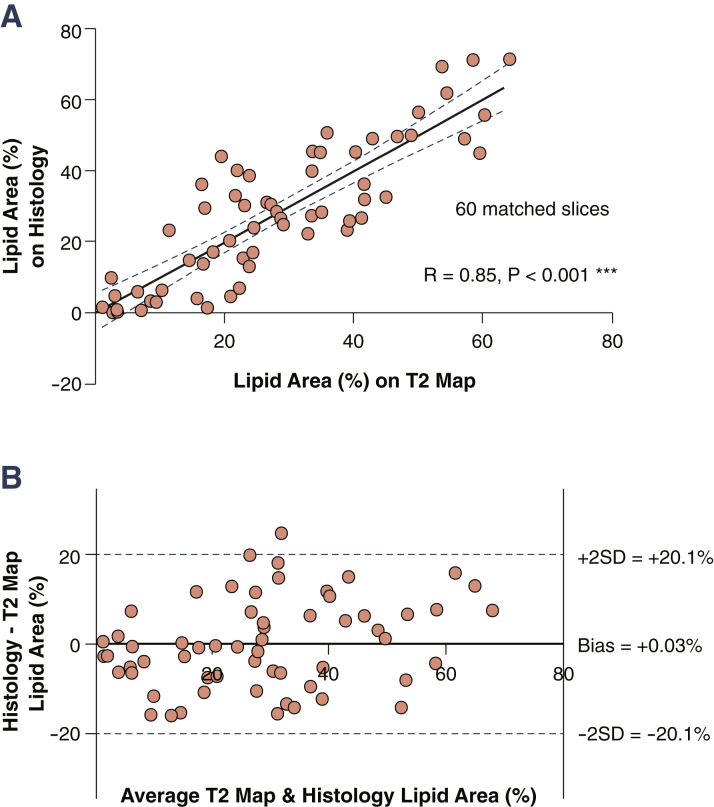
Correlation of Lipid Area Between T_2_ Mapping and Histology **(A)** Scatter plot of lipid area (%) by slice location showed excellent correlation (R = 0.85, p < 0.001). **(B)** Bland-Altman analysis showed good agreement between histology and T_2_ map with minimal bias.

We next used T_2_ maps to quantify plaque lipid content (Figure 2). Varying according to the length of each carotid atherosclerotic lesion, an average of 2.3 slices of T_2_ map data were obtained per plaque, yielding 60 matched slices. Using leave-1-out cross-validation, the combination T_2L_ = 42 ms and T_2H_ = 90 ms produced maximum R = 0.85 (p < 0.001) against lipid area (%) measured on histology (Figure 3A), thus achieving optimal T_2_ map segmentation for lipid quantification. Additionally, root mean square error was minimum (10.5%) and Bland-Altman analysis confirmed a high degree of agreement between T_2_ maps and histology, with only 0.03% underestimation on T_2_ maps (Figure 3B).

**Figure 4:**
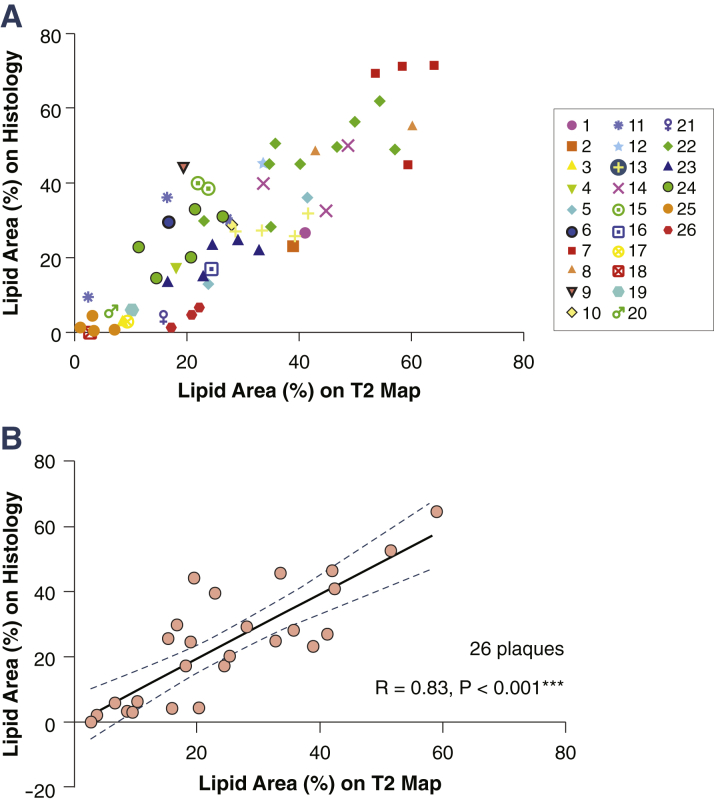
Plaque-by-Plaque Correlation of Lipid Area **(A)** Scatter plot of lipid area (%) to show slices from the same plaque (individually colored, see legend) tend to contain similar proportions of lipid. **(B)** Strong correlation maintained with plaque-by-plaque comparison (R = 0.83, p < 0.001). ***p < 0.001.

For technical validation, we made multiple comparisons of T_2_ maps and tissue stains per slice. However, for biological applications, the unit of analysis would be per plaque rather than per slice. Grouping individual slice locations from each plaque on the same scatter plot, we showed that data points from the same plaque were relatively closely clustered together (Figure 4A), indicating that slices from the same plaque tend to contain similar proportions of lipid. Furthermore, comparing the average lipid area (%) per plaque measured by T_2_ map and by histology maintained a strong correlation (R = 0.83, p < 0.001) (Figure 4B).

### Symptomatic versus asymptomatic

**Figure 5:**
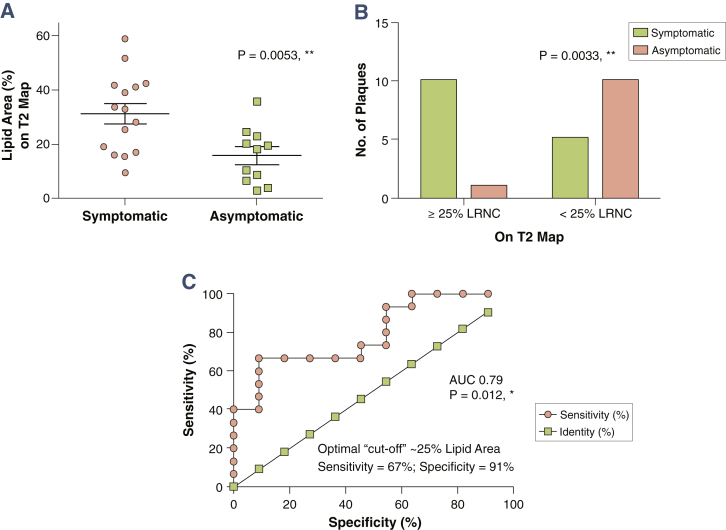
Symptomatic Plaques Contain More Lipid Than Asymptomatic Plaques **(A)** Symptomatic plaques contained significant more lipid than asymptomatic plaques (31.5 ± 3.7% vs. 15.8 ± 3.1%; p = 0.005) despite similar degree of luminal stenosis. **(B)** Chi-square test using an established cutoff of >25% to define a large lipid-rich necrotic core (LRNC) showed similar results. **(C)** Receiver-operating characteristic curve analysis showed that T_2_ mapping has a fair to good ability to discriminate between symptomatic and asymptomatic plaques. ∗p < 0.05, ∗∗p < 0.01. AUC = area under the curve.

We then tested using T_2_ mapping whether LRNC size varied between different clinical presentations. Symptomatic plaques were found to have significantly higher lipid area (%), compared to asymptomatic plaques (31.5 ± 3.7% vs. 15.8 ± 3.1%; p = 0.005, relative difference 99.4%) (Figure 5A). This was despite similar degree of carotid stenosis (81.3 ± 2.5% symptomatic vs. 84.1 ± 3.0% asymptomatic; p = NS), and only a modest difference in terms of plaque volume (128.0 ± 6.0 mm^3^ symptomatic vs. 105.6 ± 9.4 mm^3^ asymptomatic; p = 0.04; relative difference = 21.2%). In addition, using an established definition of large LRNC as ≥25% of cross-sectional area 3, 19, we further confirmed that symptomatic plaques had significantly larger LRNC using T_2_ mapping alone (p = 0.003) (Figure 5B). Finally, the receiver-operating characteristic (ROC) area under the curve was 0.79 (p = 0.01), indicating that lipid quantification by T_2_ mapping has a good ability to discriminate between symptomatic and asymptomatic plaques in our clinical cohorts. ROC analysis determined the optimal cutoff for LRNC between clinical cohorts to be ∼25% (sensitivity = 67%, specificity = 91%) (Figure 5C).

### Intraplaque hemorrhage

**Figure 6:**
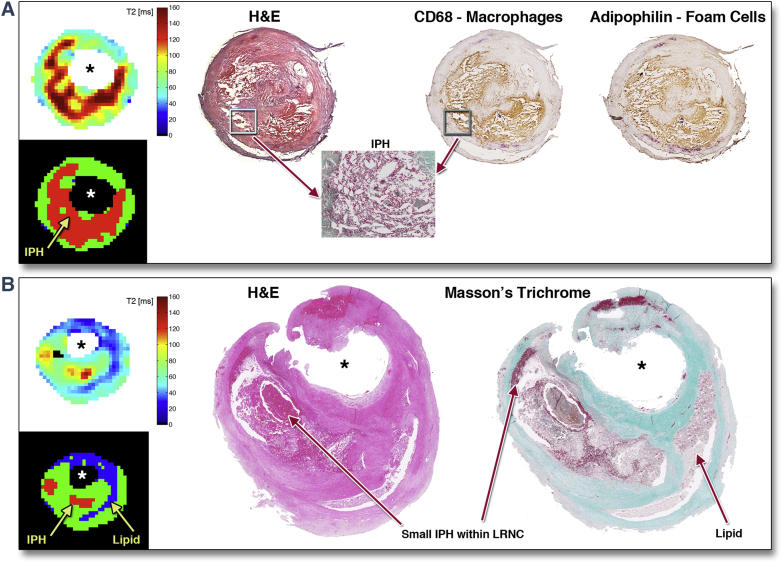
IPH Is Structurally Integral to the LRNC **(A)** Large intraplaque hemorrhage (IPH) in background lipid-rich necrotic core (LRNC). Erythrocytes and fibrin staining on Masson’s trichrome confirmed IPH. Copresence of macrophage and lipid droplets confirmed by immunohistochemistry against cluster of differentiation 68 (CD68) and adipophilin (35). **(B)** Small IPH areas, structurally continuous with background LRNC, detected by T_2_ mapping and histology. Note: a small area of fresh blood at the specimen border seen at 12 o’clock position is a surgical artifact. H&E = hematoxylin and eosin.

To clarify whether the inclusion of IPH in our calculation with a second T_2_ threshold had influenced our results, we examined the prevalence of IPH and its contribution to the total lipid area (%). Sixteen of the total 60 slices contained significant (>5%) IPH. Of these, only 7 had IPH infiltrating >50% of the lipid core. Thus IPH made a relatively minor contribution to the total lipid measurement. Figure 6 shows the copresence of IPH and lipid-filled macrophage foam cells. We then reanalyzed our data using a single T_2_ threshold of <42 ms for segmentation and lipid area calculation, by so doing effectively excluded IPH (long T_2_) as part of the LRNC in T_2_ map segmentation, yet the strength of correlation was similar to our dual T_2_ threshold approach (R = 0.79 vs. 0.85). In fact, if we excluded those 7 slices with large (>50%) IPH from the cohort, the correlation of the remaining 53 slices using a single threshold (<42 ms) approach went back to R = 0.85.

### Scan-rescan reproducibility

One of the 10 carotid plaques scanned to evaluate reproducibility was rejected due to low image quality during rescan. For the remaining 9 plaques, lipid core measurements ranged from 5.7% to 36.6% with a mean of 20.3%. The scan-rescan reproducibility was excellent (intraclass correlation coefficient = 0.89; 95% confidence interval: 0.59 to 0.98; coefficient of variation = 8.9% [plaque-by-plaque comparison]).

## Discussion

This work makes 2 principal findings. First, in vivo T_2_ mapping, on a voxel-by-voxel basis, and at high resolution, enables accurate and highly reproducible quantification of plaque lipid, validated with histology. Second, lipid quantification by T_2_ mapping can distinguish recently symptomatic plaques with high sensitivity and specificity.

Deposition and retention of cholesterol-rich lipoproteins is a cardinal feature of atherosclerosis, occurring from the earliest foam cell lesions through to the complex plaques of advanced disease. Where the rate of deposition exceeds the rate of clearance, cholesterol and cholesterol derivatives can accumulate within the plaque. The presence of a large LRNC (especially associated with a thin overlying fibrous cap) has been associated with high risk of rupture and atherothrombotic complications (3). Furthermore, the presence of lipid in the plaque is a driver of local inflammation (20) and altered gene expression (21). Because of these central roles, quantification of plaque lipid has been a key aim of various atherosclerosis imaging techniques such as intravascular ultrasound (22), near infrared spectroscopy (23), cardiac computed tomography (24), and vascular magnetic resonance imaging (11).

Since the first description of Toussaint et al. (25), magnetic resonance techniques have been applied for the detection of LRNC 26, 27. However, a significant limitation of this approach is low sensitivity, such that LRNC can be detected only in a minority of plaques with relatively large coalesced lipid pools. In a survey of 100 patients with varying extents of coronary atheroma, only 20% had a discernable LRNC in the carotid artery on multicontrast CMR (28). Reliable detection of small scattered pools of lipid depends on image resolution and signal-to-noise ratio in both T_2_ mapping and multicontrast CMR. The main advantage of T_2_ mapping is to provide quantitative information, on a per-voxel basis, using direct measurement of an absolute physical property of plaque components. Our approach to lipid quantification is therefore more objective and less dependent on acquisition parameters than multicontrast CMR, which relies on relative signal intensities for plaque segmentation. In addition, T_2_ mapping does not suffer from blurring artifacts intrinsic to fast spin echo acquisition (12), nor does it require image coregistration and intensity correction (13). Finally, it is more time efficient (one-fifth of the time required by multicontrast protocol for LRNC quantification) 6, 11 and without exposing patients to a gadolinium-based contrast agent. Because the validation required tissue obtained by endarterectomy (undertaken for severe stenotic disease), our series contains relatively large lesions (of AHA classes IV to VIII on histology). In this context, plaque lipid was detectable in virtually all cases.

Atherosclerosis regression is a term used to denote favorable remodeling of plaque, for instance in response to aggressive reduction in plasma level of atherogenic lipoproteins (29). Normalization of plasma lipid profile in genetically modified hyperlipidemic mice with atherosclerosis leads to marked loss of plaque lipid and deposition of fibrous tissue (7), with similar changes inferred in man (30). This has important implications in the clinic because some antiatherosclerotic agents may well change plaque lipid content, without necessarily altering total plaque volume. This suggests that accurate quantification of plaque lipid might prove useful for patient stratification and selection for intensive lipid lowering therapies or monitoring of response to treatment, including in clinical trials. The technique validated here shows excellent scan-rescan reproducibility and would be well suited to serial application in lipid-intervention trials. This may be important since several new classes of lipid lowering therapies have been developed, including lipoprotein(a) and apolipoprotein B antisense, microsomal triglyceride transfer protein inhibition, cholesteryl ester transfer protein inhibitors, and proprotein convertase subtilisin/kexin type 9 inhibitors, but considerations of cost effectiveness, mode of administration, and, in some cases, potential toxicity mean that they are unlikely to be used indiscriminately in patients with atherosclerotic disease. Methods for rational stratification are therefore needed.

Applying T_2_ mapping in our different clinical cohorts, we found that despite having similar degree of carotid stenosis, and only a modest 21.2% difference in terms of plaque volume, symptomatic plaques had 99.4% larger LRNC than asymptomatic plaques. ROC analysis confirmed our technique having good ability to distinguish between symptomatic and asymptomatic plaques with optimal LRNC cutoff at 25% of the cross-sectional area, in agreement with large-scale histological series in both carotid (3) and coronary (31) locations. The ability to accurately quantify plaque lipid might also prove useful in informing decisions between carotid surgery and stenting because a large LRNC is considered to be an important factor determining the amount of stent-related downstream debris (32) and has been shown to increase risk of stroke following carotid stenting 32, 33. Three-dimensional rendering of lipid distribution in relation to local vessel anatomy using quantitative T_2_ mapping data may assist interventional procedural planning to minimize such risks (Figure 2, right panels).

### Study limitations

The main limitation of our method is the sensitivity to motion artifacts, a fact reflected in the scan rejection rate (35%), which is comparable to that of multicontrast CMR (30%) (11). Strategies for motion correction are currently being actively pursued. Another limitation is the challenge to measure IPH independently, mainly because of the tendency for hemorrhage to be mixed with LRNC. Moreover, as IPH ages and organizes, not only do its magnetic resonance properties change (16), but also its histological appearance, as organization replaces fibrin with a more collagen-like extracellular matrix structure (34). We therefore analyzed our data using 2 approaches: 1 with dual T_2_ thresholds that included both lipid and IPH and another with a single T_2_ threshold to effectively exclude IPH as part of the LRNC measurement. By either method, the strength of correlation was similar (R = 0.79 vs. 0.85). This demonstrated empirically that T_2_ mapping does track plaque lipid content accurately, whether or not IPH is considered as part of the LRNC calculation. Finally, this histological validation study has a relatively small number of subjects and CMR slices. To utilize the available data most efficiently, we employed leave-1-out cross-validation to test the performance of our T_2_ segmentation model on data that were not used to build it.

## Conclusions

We provided histological validation of a new in vivo multislice T_2_ mapping technique for quantification of plaque lipid content. Not only can T_2_ mapping distinguish between symptomatic and asymptomatic plaques on the basis of their lipid content, but we showed that despite similar degree of luminal stenosis and only modest difference in plaque volume, symptomatic plaques contained nearly double the lipid content compared with asymptomatic plaques on T_2_ maps. This technique may be of value in patient selection and evaluating response to new antiatherosclerotic treatment and interventions.

Perspectives **COMPETENCY IN MEDICAL KNOWLEDGE:** Quantitative T_2_ mapping is a novel technique in characterization of carotid atherosclerotic plaques. Using histological validation, this study demonstrated its ability to differentiate various plaque components, with an emphasis on lipid quantification. Symptom-related plaques were shown to contain significantly more lipid despite similar degree of luminal stenosis and only modest difference in plaque volume compared with asymptomatic plaques. T_2_ mapping is accurate and reproducible; it is an alternative technique to the conventional multicontrast approach, which may be of value in future lipid intervention trials and cardiovascular risk stratification.

**TRANSLATIONAL OUTLOOK:** Technical improvements are actively pursued, to reduce imaging time by compressed sensing, increase spatial resolution coverage and signal-to-noise ratios by better multichannel coils, and reduce motion artifacts by the development of image navigator–based approaches. How the changes in the size of lipid-rich core in vulnerable plaques, as an imaging biomarker, translate to the modification of cardiovascular risks remain to be elucidated.
