# Supplementary material for: Quantification of Lipid-Rich Core in Carotid Atherosclerosis Using Magnetic Resonance T2 Mapping: Relation to Clinical Presentation
Source: JACC Cardiovasc Imaging. 2017 Jul;10(7):747–56. doi: 10.1016/j.jcmg.2016.06.013 (PMC5502905; doi:10.1016/j.jcmg.2016.06.013)

**Supplementary Methods**

*MRI acquisition protocol*

Time-of-Flight (TOF) angiography of the carotid arteries was acquired using 3D Fast Low Angle Shot (FLASH) with flip angle = 25°, TR = 20 ms, TE = 3.3 ms, FOV = 200 ×150 mm^2^, matrix size = 384 × 288 and slice thickness = 1 mm. 74 TOF consecutive slices were imaged in order to localize the carotid bifurcation and the atherosclerotic plaque. Multi-slice carotid T_2_ maps were generated from 14 images with TE = 9 - 127 ms acquired using the DANTE-MESE sequence, which combines black-blood preparation based on non-selective Delay Alternating with Nutation for Tailored Excitation (DANTE) pulse trains with chemical-shift-selective fat saturated Multi-Echo Spin-Echo (MESE) sequence. MESE parameters were TR = 2000 ms, TE = 9 - 127 ms, partial Fourier = 5/8, FOV = 128 × 128 mm^2^, matrix size = 384 × 384, slice thickness = 2 mm and slice gap = 2 mm. DANTE parameters were FA = 8°, number of pulses = 120, interval between pulses = 0.5 ms, Gz = 18 mT/m and gradient duration = 0.4 ms. DANTE-MESE acquired 5 slices in 4 minutes and was then repeated consecutively with a 2 mm off-set resulting in a total coverage of 10 slices of 2 mm thickness, i.e. 2 cm of the target carotid artery in 8 minutes. Patient head movement was limited by a head holder and they were instructed not to move and to try not to swallow during each acquisition.

**Supplementary Figure Legend**

Supplementary Figure 1 *Intraplaque haemorrhage and LRNC.* Large lipid rich core with evidence of fresh intraplaque haemorrhage from plaque rupture. Blood entered lipid core under pressure histologically *indistinguishable* from rest of LRNC on Oil red O as haemorrhage infiltrated deep within lipid core.


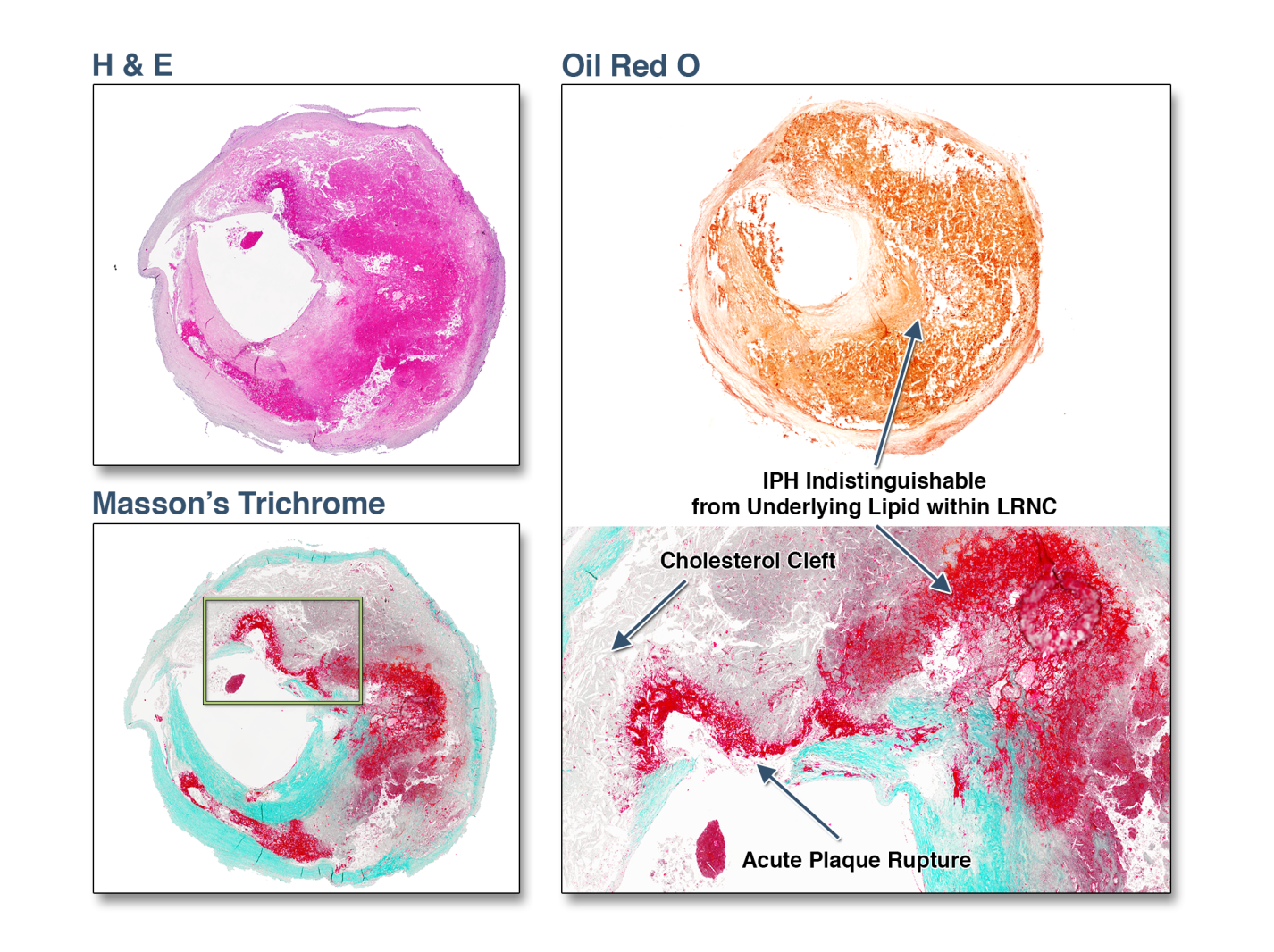

Supplement: Online Appendix and Online Figure 1 [file mmc1.docx]
